# From the New Diagnostic Criteria to COVID-19 Pandemic Passing Through the Placebo Effect. What Have We Learned in the Management of Pediatric Migrane Over the Past 5 Years?

**DOI:** 10.3389/fneur.2022.935803

**Published:** 2022-07-13

**Authors:** Laura Papetti, Samuela Tarantino, Fabiana Ursitti, Romina Moavero, Martina Checchi Proietti, Giorgia Sforza, Gabriele Monte, Michela Ada Noris Ferilli, Martina Balestri, Federico Vigevano, Massimiliano Valeriani

**Affiliations:** ^1^Pediatric Headache Center, Department of Neuroscience, Bambino Gesù Children Hospital, Istituto di Ricovero e Cura a Carattere Scientifico (IRRCS), Rome, Italy; ^2^Child Neurology and Psychiatry Unit, Tor Vergata University of Rome, Rome, Italy; ^3^Child Neurology Unit, Bambino Gesù Children's Hospital, Istituto di Ricovero e Cura a Carattere Scientifico, Rome, Italy; ^4^Center for Sensory-Motor Interaction, Aalborg University, Aalborg, Denmark

**Keywords:** migraine, headache, children, treatment, management, diagnosis

## Abstract

In 2018, the Food and Drug Administration (FDA) approval of anti-calcitonin gene-related peptide (CGRP) therapies for the treatment of migraine represented a milestone for the management of the disease in adults. On the contrary, the novelties in the field of pediatric migraine are inserted in a different scenario and still concern: (1) diagnostic criteria of the international classification of headache disorders-3 (ICHD-3) that show numerous limits of applicability in the developmental age; (2) the release of the results of the Childhood and Adolescent Migraine Prevention (CHAMP) study that raised doubts about the usefulness of traditional drugs for the treatment of pediatric migraine; (3) the Coronavirus disease 2019 (COVID-19) pandemic has put the spotlight on the importance of managing the psychological factors associated with the disease. In this mini review we discuss the most relevant news in pediatric migraine over the last 5 years.

## Introduction

Headache is a very frequent symptom in children, with a higher incidence of primary forms including migraine. The prevalence of migraine in children ranges from 3 to 20% but tends to increase in adolescence (1). Migraine can become a very disabling condition due to the intensity and frequency of the attacks with an impact on the quality of life of the child and his family. About 2–5% of children with migraines may develop chronic migraines over time (2). In these cases, migraine significantly interferes with the child's activities with a reduction in school performance and social relationships (2–4).

Migraine may be improved by pharmacological prophylaxis and non-pharmacological treatment, such as lifestyle modifications and complementary therapies (5–7). The established management of migraine in children and young people starts with the clinical suspicion, then diagnosis of migraine, followed by information and advice, such as lifestyle changes, non-drug treatments, and shared decision-making about medication (1, 3). Much of this assessment, diagnosis and treatment have not changed over the last years.

In the last 5 years, three scenarios had an impact on the management of pediatric migraine and these are: the publication in 2017 of the Childhood and Adolescent Migraine Prevention (CHAMP) study which raised the question of which therapy is most efficacious for children (8); second in 2018, the release of the third version of the international classification of headache disorders (ICHD-3) that includes the diagnostic criteria for migraine (9) and finally from 2020 to date the Coronavirus disease 2019 (COVID-19) emergency which had many repercussions on the management of chronic diseases including migraine (10).

The purpose of this review is to focus on fundamental topics regarding the management of migraine in pediatric age, discussing the data that have emerged from the scientific landscape in the last 5 years and the impact that the aforementioned events had on pediatric migraine. To this end, we interviewed the managers of the main Italian pediatric headache centers on these topics. Their views along with literature data were used to carry out this mini review.

## Methods

We made this mini review through a literature review carried out on PubMed using the original articles published in the last 5 years about migraine in patients aged 5–18 years. We revised original article on general management, diagnosis, and treatment. Through this review, we have intercepted the three most important events in this time range and they are: the publication of version 3 of the IHS diagnostic criteria, the publication of the CHAMP trial, and the COVID-19 pandemic. We then started from these three events to question the managers of the main headache centers for children and adolescents (see acknowledgments) in Italy through a questionnaire with open answers. The purpose of the questions summarized in Table 1 was to ask their opinion on the present scenario regarding juvenile migraine in particular for diagnosis and treatment.

**Table 1:** List of questions of the interview.

**General questions**
1. In the last 5 years, in what area has significant news emerged on migraine in children and adolescents?
2. Do you think that the COVID-19 pandemic has led to changes in migraine management?
3. What do you think the priorities on management of pediatric migraine to be addressed in future studies?
**Diagnosis**
1. The new headache classification system according to ICHD-3 has helped to improve the diagnosis of migraine in children and adolescents?
2. What are the main difficulties in the migraine diagnostic process?
3. What kind of intervention could improve the diagnostic workout (for example, information/education of families and general practitioners or other)?
**Therapy**
1. In your opinion, has the CHAMP study changed the way we treat migraine in children and adolescents?
2. What aspects must be considered when choosing the treatment?
3. What aspects would need to be investigated for the study of migraine treatment in the developmental age?
4. How do you think the use of anti-CGRP monoclonal antibodies will modify migraine therapy in children and adolescents?
**Outcome**
1. In your opinion, which factors contribute most to a poor prognosis of migraine?
2. What type of intervention could improve the outcome of the migraine (for example, information/education of families and general practitioners or other)?

Results are summarized in Table 2.

**Table 2:** Summary of the main results of the study.

**Diagnosis**
Adult ICHD-3 criteria are used for the diagnosis of migraine in children with some exceptions (red):
A. At least five attacks 1 fulfilling criteria B–D
B. Headache attacks lasting 2–72 h
C. Headache has at least two of the following four characteristics:
1. Unilateral o bilateral location
2. Pulsating quality
3. Moderate or severe pain intensity
4. Aggravation by or causing avoidance of routine physical activity (e.g., walking or climbing stairs)
D. During headache at least one of the following:
1. nausea and/or vomiting
2. photophobia and phonophobia
E. Not better accounted for by another ICHD-3 diagnosis.
**Traditional pharmacological therapy**
The CHAMP trial found a high placebo response rate without differences between amitriptyline and topiramate.
Placebo response observed in the trails can be influenced by patients, caregiver's expectation, natural course of disease, and parallel interventions.
The true placebo effects should be investigated in trial with an untreated group.
The indication of FDA for the use of topiramate for the treatment of episodic migraine in adolescents 12–17 years of age still remains.
Numerous data efficacy data from the previous trials on the use of traditional drugs for treatment of migraine in children and adolescents are available.
**The experience of Lockdown and COVID 19 pandemic**
The COVID-19 emergency highlighted how telemedicine can be a support tool for the management of migraine patients.
The improvement of the migraine course during the first lockdown of March 2022 underlined how stress management is essential for children and adolescents (anxiety, school stress, and coping strategies)
**Future perspective**
Recent data underline the usefulness of cognitive behavioral therapy for the treatment of juvenile migraine, alone, or in combination with drug therapies.
Preliminary data on the use of CGRP antibodies for migraine in adolescents show promising results.

## Diagnosis

The “International Headache Society” released the ICHD-3 in 2018 (9). As this version is based on a large body of research on headache, in contrast to the previous editions that were mostly based on opinion of experts, it is being considered as a major step forward in the diagnosis and management of headache. The important ones include changes in some terminologies, addition of new categories and changes in diagnostic criteria (9). Unfortunately for the diagnosis of migraine in pediatric age, the ICHD-3 still does not have a section dedicated to childhood but clarifications are made in some notes of the criteria dedicated to adults (9). Pediatric migraine experts agree that the criteria continue to have different limits when applied to developmental age with the possibility of escaping up to 40% of diagnoses (11, 12). One of the most discussed points of the ICHD-3 is having again brought the minimum duration of the migraine attack to 2 h and no longer to 1 h as in the previous version (13). The authors state that the evidence for untreated durations of <2 h in children has not been substantiated (13). However, this contrasts with the data reported by various authors who argued that the duration of the migraine attack can be much <2 h (12, 14). Several studies have shown that the reduction of the lower limit of the attack to 1 h as reported in the second version of ICHD, had greatly improved the sensitivity of the criteria (14–16). Another point of discussion regards the localization of pain. In children, pain is often bilateral and the most common location of pain is frontal (60.9%), whereas it is ocular (53.17%), followed by temporal (38.67%) in adults (17). The quality of pain in children is usually constricting or pounding, whereas in adults it is a frequent pulsating (18).

In very young children, the diagnosis of migraine can be particularly challenging due to their inability to verbalize symptoms, such as photophobia or phonophobia. Reports are commonly based on parent or caretaker's observations, which are often at the core of diagnosis. Diagnostic criteria for younger children should emphasize behavioral aspects rather than verbal reports. All these arguments underlined the necessity to provide a dedicate section of criteria for diagnosis of migraine in children (19).

The criteria also present some limitations in the differential diagnosis between primary and secondary headaches. In fact, for each form of headache the criterion “not better accounted for by another ICHD-3 diagnosis” is included (9). However, this creates confusion in situations such as patients with a history of previous migraine who then develop worsening in some circumstances such as infections or trauma (20). Furthermore, in centers specialized in the management of headaches, children with migraines often arrive years after onset, and after the management of the headache has been by general pediatricians and in extreme cases by parents. This often involves confusing migraine symptoms with other conditions such as refractive vision defects, sinusitis, allergies, or food intolerances. Although there has been increased recognition of migraines in children in recent years, it still remains under-recognized and underdiagnosed (20).

Furthermore, the territorial realities are very different, and a child and his family do not always have the opportunity to contact specialized headache centers. It may be in this sense that it would be useful to improve the information of general doctors and neurologists who deal with adult headaches as well as general pediatricians (21). This factor was further emphasized in the COVID-19 era when many headache centers were closed due to the emergency and the lockdown (22, 23).

Among the advantages of the ICHD-3 criteria was that of highlighting the periodic syndrome related to migraine that are called “episodic syndromes that may be associated with migraine” (9, 24). In addition to the already known benign paroxysmal vertigo of childhood, cyclical vomiting and abdominal migraine, another condition has been added that is the benign paroxysmal torticollis (9). Cyclical vomiting and abdominal migraine have been clubbed together as “Recurrent gastrointestinal disturbance” (9, 24). Since it is frequently described that children with migraine also have a history of episodic syndromes, consideration should be given to including the latter among the criteria for the diagnosis of migraine in the child (24).

Improving the diagnostic process of migraine in children and adolescents is essential is crucial milestone and opens the door to effective treatment.

## Therapy

In recent years, the treatment of migraine in adults has been revolutionized by the use of monoclonal antibodies against CGRP and its receptor (25–27), and serotonin receptor 5-HT1F agonists (28). However, randomized controlled trials (RCTs) on these new drugs in children and adolescents are ongoing (29), and to date, the treatment of both prophylaxis and the attack in this group remains firm to the evidence of the old traditional drugs used in adults (5). The most important novelty in juvenile migraine was the publication in 2017 of the results of the CHAMP study in the New England Journal of Medicine. This trial compared amitriptyline, topiramate, and placebo in 328 patients of youth ages 8–17 with migraines. The authors found that all groups underwent to the reduction of frequency of the attacks or headache-related disability over a period of 24 weeks without significant differences between placebo and active drugs. The two medications had greater side effects than the placebo. The conclusion was that the risk-to-benefit profile of the two most commonly used preventive medications does not suggest their use as first-line intervention choices for migraine in children and adolescents (8). Later, a survey study, conducted as follow-up to the CHAMP trial, found that children and adolescents with long-standing migraine experienced and maintained meaningful improvements in headache status for up to 3 years after treatment. In this study, headache days and disability improved with most participants reporting no medication. No statistically significant differences were found between the CHAMP and treatment groups. Given this, meaningful clinical improvement does not appear to be associated with the pharmacological action of preventive medication but rather by other mechanisms, such as the expectation of response or fluctuations of disease severity over the lifespan that may occur for some individuals with a recurrent pain condition (30).

Some authors have emphasized the limitations of the CHAMP study such as the long follow-up period, the behavioral interventions offered to the placebo group, and the exclusion of certain subgroups such as the most severe migraines and younger children (5, 31–35). Moreover, real-word data on the efficacy and safety of traditional drugs for the pediatric migraine published both before and after the CHAMP study have not been canceled (32–34, 36). For example, before the release of the CHAMPS results, the FDA-approved topiramate for patients aged 12 years and older with migraine and at present, this approval still stands (36). In addition, in several studies, amitriptyline alone or in combination with non-drug therapies has also shown efficacy and safety for the treatment of migraine in children (37–39). Conversely, recent meta-analysis on the efficacy of preventive medication for migraine was published and corroborated the CHAMP findings (40, 41).

The CHAMP study (8) and the follow-up survey (30), actually highlighted an open question for years, namely that the treatment of migraine in children is not simply a treatment for adults at lower doses, but must be a therapy created on the model of the child with migraine. This has opened a heated debate among researchers dedicated to the treatment of pediatric migraine (5, 31, 42–44). Future research should investigate whether neurobiological or pain processing changes, functional changes in brain activity, psychological factors, or treatment expectations can result in different responses to a specific treatment. Another important topic is to analyze fluctuations of migraine attack frequency over time and determine the most clinically relevant length of probable prophylactic treatment. Finally, we need to accept that placebo is not a null response and investigate what creates this response from a clinical and pathophysiological viewpoint. In fact, there is indirect evidence that the placebo effect is more pronounced in children and adolescents than in adults (35).

We often and wrongly equate the response seen in the placebo arm of a clinical trial with the placebo effect. The perceived placebo effect is the consequence of the interaction between different factors, like natural course of illness, expectations of patients and caregivers, parallel intervention, or other time effect (45).

The quantification of the placebo effect would, therefore, require comparison with a non-treated group, which is rarely included in clinical trials. If the placebo effect is confirmed to be large in children and adolescents, innovative treatment strategies should be considered that harness the placebo effect in the treatment of juvenile migraine (35, 40, 46).

From a clinical point of view, what alternatives do we have to drug therapies? We are eagerly awaiting the possibility of using CGRP monoclonal antibodies (CGRP mAb) for migraine in children. However, their use is currently limited in phase 3 trials. These concerns erenumab (OASIS), galganezumab (REBUILD-1), fremanezumab (AJOVY) and eptinezumab (PROSPECT-2) (29).

Phase 1 studies in pediatrics have to date documented a safety profile of CGRP comparable to that of adults (47, 48). A retrospective study was recently published on adolescents with chronic migraine who received at least one dose of CGRP mAb. This study showed that the CGRP monoclonal antibody treatment appears to benefit a proportion of adolescents with chronic refractory headache disorders (49).

While we wait for the results of the CGRP mAb trials what alternatives do we have to prophylaxis with traditional drugs? What recent data do we have on the use of non-pharmacological therapies?

Among these, the use of nutraceuticals (50, 51), onabotulinumtoxinA (52, 53), and psychological therapies (54–56) has attracted a lot of interest in last years.

Regarding nutraceutical options among the most recently studied molecules are polyunsaturated fatty acids (PUFAs) (49) and palmitoylethanolamide (PEA) (51). However, these studies lack of controlled data and involve a small number of patients. Although the efficacy data are not conclusive, they have excellent tolerability profiles with few side effects (57).

In a double-blinded placebo-controlled trial, subjects aged 8–17 years old diagnosed with chronic migraine received OBTA treatment with protocol consisting of 155 units at 31 injection sites in 3-month intervals. Subjects reported a significant decrease in the frequency and intensity of migraines with a reduction of the PedMIDAS score, that is, increased functionality from baseline values compared with the placebo group (53). The results of this study contrast with those of a previous trial published a year earlier in which the effectiveness of OBTA was comparable to that of placebo (52). Both studies show that OBTA treatment is still safe even in children and adolescents even if in younger patients, the injection mode may be less tolerated (51, 52).

Evidence for the efficacy of psychological interventions for the treatment of headaches in youth has grown substantially over the past several decades (55, 56, 58–60). There is a strong and growing body of evidence demonstrating the effectiveness of psychological approaches, primarily cognitive-behavioral therapy (CBT), for treating migraine in children and adolescents (37, 38, 54, 55). Results from a large meta-analysis including 14 RCTs supported CBT as an effective form of treatment for juvenile headache conditions as compared to placebo, waitlist, or medication, producing clinically significant improvement in headache frequency (a 50% or greater reduction in headache frequency). Moreover, the efficacy was maintained long-term (54). Further, a 2018 Cochrane review of psychological therapies for the treatment of chronic pain in children and adolescents found that cognitive and/or behavioral interventions significantly reduced the headache days and intensity across 15 RCTs (55).

Some pilot studies demonstrated the acceptability and feasibility of a mindfulness-based treatment for adolescents with recurrent headaches (59, 60).

Collectively, these results highlighted that non-medicine interventions for the treatment of migraine in youth are safe and effective. Further optimization of available psychological interventions is needed, and focus should be placed on addressing the impact of headaches on the daily functioning and quality of life of children and adolescents.

To date, we have no efficacy data in children and adolescents for other non-drug therapies such as non-invasive vagal nerve stimulators or non-invasive neuromodulator techniques.

To resume, although much has been said about it in the CHAMP study, the real role of the placebo effect in migraine remains to be defined. We also have efficacy data on the use of CBT which must, therefore, be considered in the treatment of juvenile migraine. However, these data suggest that the effects of CBT may begin to manifest several months after the start of treatment (38).

In addition, CBT often has limits to its use: the experience changes from center to center; in many countries, the treatment is private with high costs for families and there is often a distrust of parents on the psychological management of the disease.

Based on this and until we have reliable data on the use of monoclonal antibodies, traditional drug therapy still finds its space and rationality. Traditional drugs should, therefore, be considered in those cases in which migraine is becoming disabled and interferes with the quality of life, when the response to attack drugs is low and when there is a risk of chronicization.

The choice of drugs must always be personalized and must take into account the present comorbidities (for example, psychological, behavioral, and related to weight and sleep disorders). From this perspective, possible side effects can sometimes become part of the treatment strategy (for example, topiramate for overweight patients or amitriptyline if a sleep disorder or mood deflection coexists).

## The COVID-19 Pandemic

The COVID-19 pandemic and above all the restriction measures imposed by the lockdown has led to two reflections on the management of migraine. The first concern is the need to emphasize the management of lifestyle factors and sources of stress such as school activity in the treatment of migraine in children and adolescents. The second concern is a new way of managing patients suffering from chronic diseases, such as migraines, which involve the use of telemedicine and the reinforcement of local structures.

The 2020 lockdown restriction measures necessary to face the COVID-19 pandemic have led to a temporary reshaping of the lifestyle of adults and children. Confined to the home, children, in particular, have undergone changes in sleep–wake rhythms, exposure to electronic devices, and reduced physical activity. An exceptional fact was the suspension of school activities. Above all, the rest from school and extracurricular activities and the reduction of academic commitment have led to a significant improvement in the headache trend. Even patients with chronic migraines or those who had not responded to drug therapies, during the 2020 lockdown experienced a significant improvement of headache course. This was also strongly correlated with a reduction in the levels of school anxiety. The improvement was independent of the geographic area of origin and pharmacological prophylaxis. These results suggested that the management of emotional and psychological factors is mandatory for the management of headache in children and adolescents (10).

As well as in adults, comorbidities with psychiatric disorders are frequent also in developmental age (61, 62). Depression is one of the most common psychiatric comorbidities in patients with migraine with a bidirectional relationship between migraine and depression being bidirectional (63). In patients with migraine disease, depression is a significant predictor of migraine evolution into chronic disorder (64). Also, anxiety may be a precipitating factor that increases the risk for headache (65, 66). Additionally, research suggests that some children may be less able to cope with daily life stressors, resulting in an increased number and severity of headaches (66).

The experience of the lockdown suggests that the clinician must always make it clear to the patient and parents that intervention on the sources of stress can be more effective than any pharmacological treatment (10).

Another aspect is that during the COVID-19 pandemic, the hospital resources dedicated to migraine have often been redeployed to COVID-19 management. Therefore, migraine consultations have been canceled or postponed. To face these changes, it became necessary to find alternative strategies for the management of migraine patients, such as telemedicine (67, 68). Treatment efforts were modified to make use of telephonic and internet communication to maintain the care of patients with headache (68). In particular, telemedicine has proved effective in verifying the response to drug treatments and the course of headaches after their suspension. Furthermore, through telemedicine, it has been possible to remodel behavioral therapy strategies such as mindfulness or psychotherapy (67, 68). Several studies prior to the COVID-19 had demonstrated the validity of telemedicine for monitoring patients with migraines (22, 69–71). This approach could be considered a first step toward a new era of patient care that maintains efficacy while conserving time and resources for both patients and providers.

Finally, due to the temporary closures of headache centers during the pandemic, general pediatricians have found themselves managing a greater number of children with headaches. This further confirmed the need to increase knowledge among general practitioners on the correct diagnosis and treatment of juvenile patients with headache. Children with primary headache, such as migraine, received often incorrect diagnoses or unsatisfactory treatments due to a lack of information by general practitioners. Education of general pediatricians on the management of headache would avoid diagnostic delays, the risk of headache worsening, and unnecessary overloading of the headache centers.

## Conclusion

Since the release of the latest version of the ICHD-3 criteria to date, a few steps forward have been made for the management of pediatric headache in terms of facilitating the diagnostic process and drug treatment. Pending the release of data on the efficacy and safety of CGRP mAb for pediatric migraine, we currently have no reliable data on the efficacy of traditional drugs. The era of the COVID-19 pandemic taught how much the intervention on the lifestyle and the management of anxiety, stress, and depression are fundamental to reach the goal of a migraine control in children as well as the information of relatives and general pediatrics can be of great help in managing headaches.

## Author Contributions

LP, ST, and MV contributed to conception and design of the article. LP wrote the first draft of the manuscript. FU, MF, GS, and GM wrote sections of the manuscript. MB and RM revised literature. All authors contributed to manuscript revision, read, and approved the submitted version.

## Conflict of Interest

The authors declare that the research was conducted in the absence of any commercial or financial relationships that could be construed as a potential conflict of interest.

## Publisher's Note

All claims expressed in this article are solely those of the authors and do not necessarily represent those of their affiliated organizations, or those of the publisher, the editors and the reviewers. Any product that may be evaluated in this article, or claim that may be made by its manufacturer, is not guaranteed or endorsed by the publisher.
